# Supplementary figures and images for: Seasonal dynamics and environmental drivers of tissue and mucus microbiomes in the staghorn coral Acropora pulchra
Source: PeerJ. 2024 May 30;12:e17421. doi: 10.7717/peerj.17421 (PMC11144401; doi:10.7717/peerj.17421)

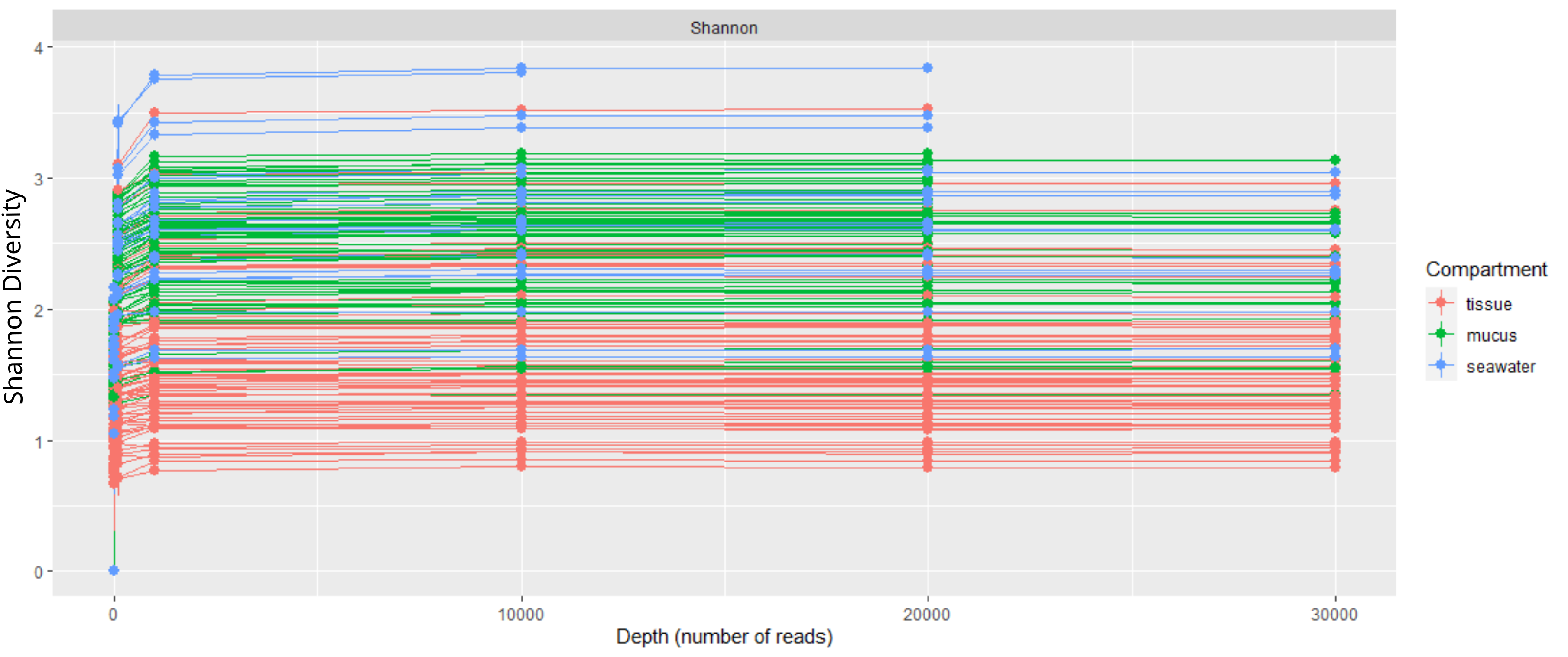

Supplement: Supplemental Information 1 — Shannon diversity plateaued at sequencing depth of approximately 1000 reads. [file peerj-12-17421-s001.png]

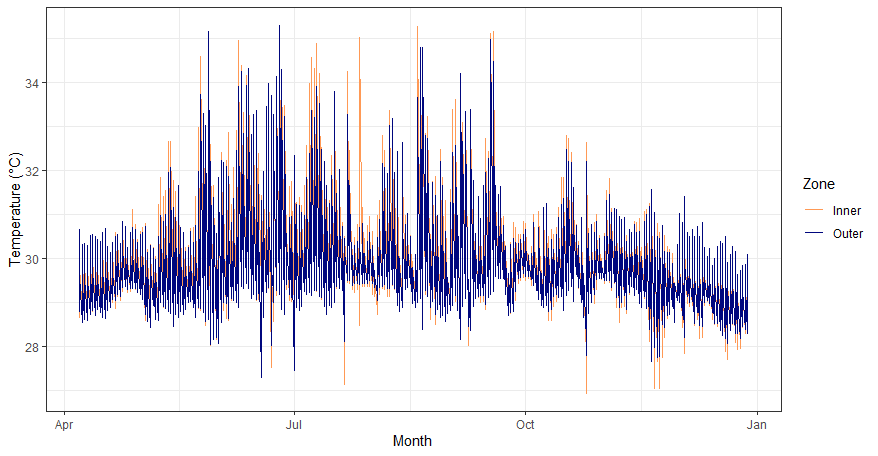

Supplement: Supplemental Information 2 — Temperatures were plotted based on their daily value. On average, the inner zone had a slightly higher temperature than the outer zone. [file peerj-12-17421-s002.png]

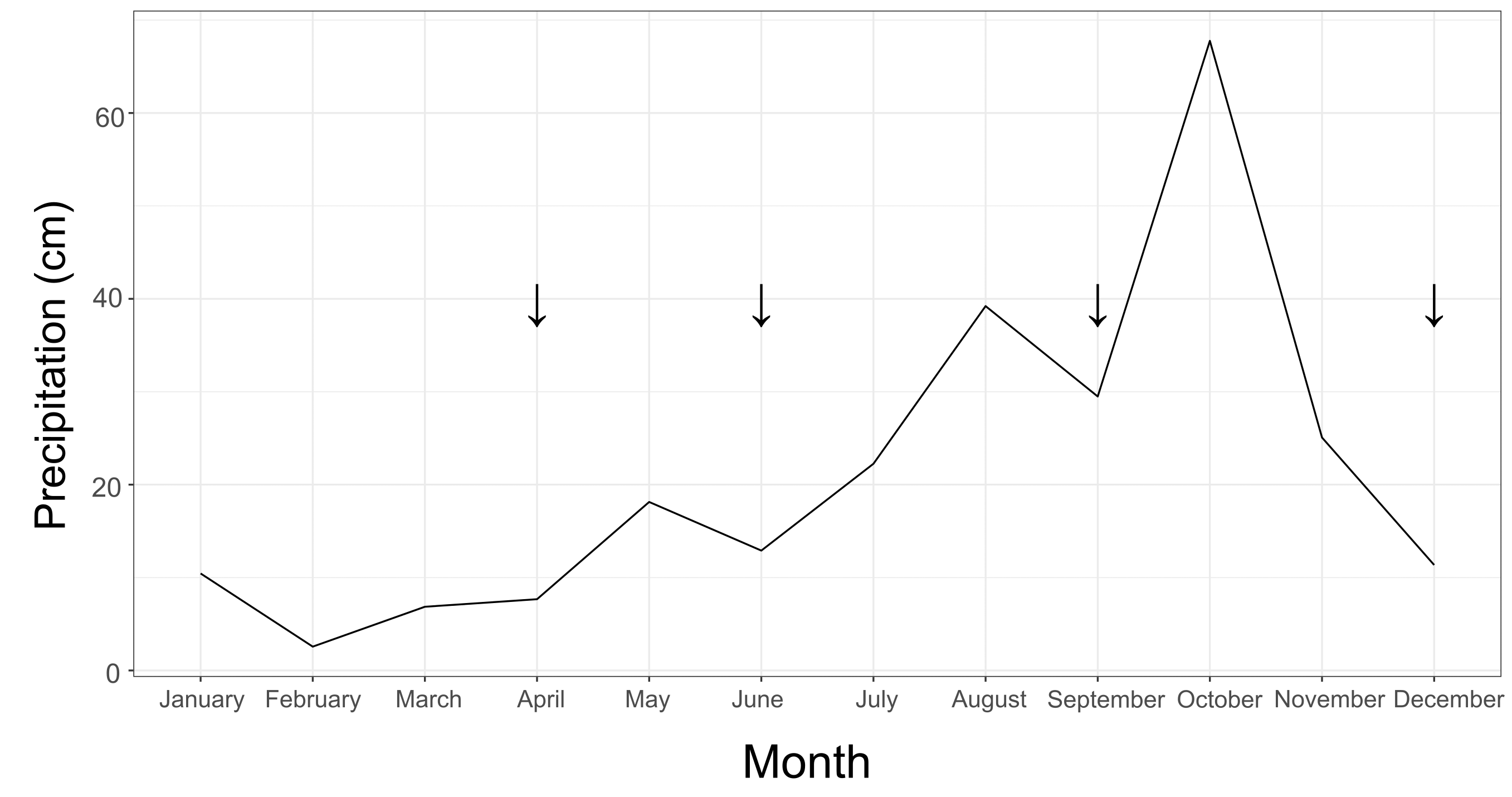

Supplement: Supplemental Information 3 — Arrows indicate sampling periods: the end of April, the end of June, the end of September, and the end of December. [file peerj-12-17421-s003.png]

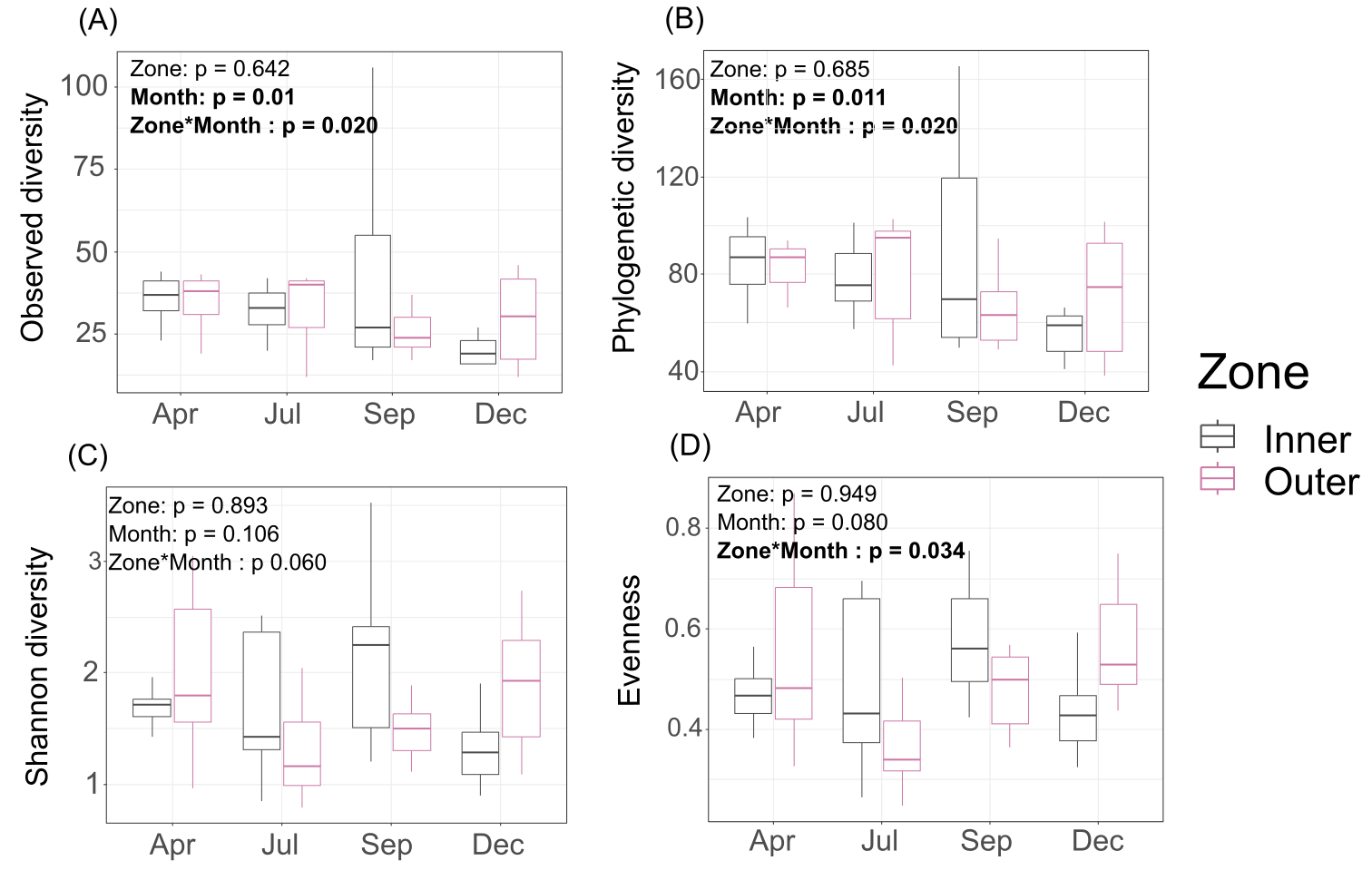

Supplement: Supplemental Information 4 — (A) Observed diversity; (B) phylogenetic diversity; (C) Shannon diversity; (D) evenness. [file peerj-12-17421-s004.png]

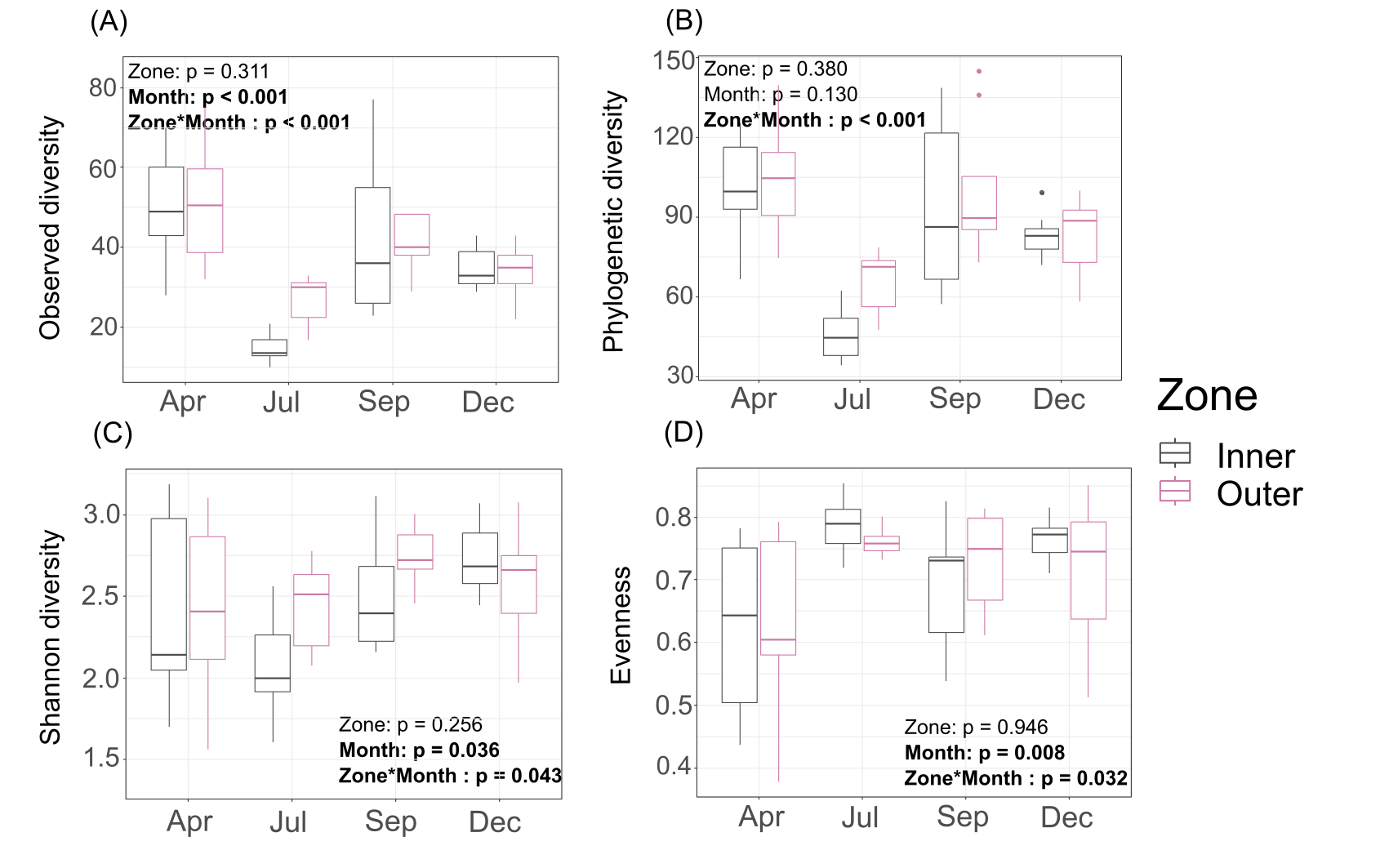

Supplement: Supplemental Information 5 — (A) Observed diversity; (B) phylogenetic diversity; (C) Shannon diversity; (D) evenness. [file peerj-12-17421-s005.png]

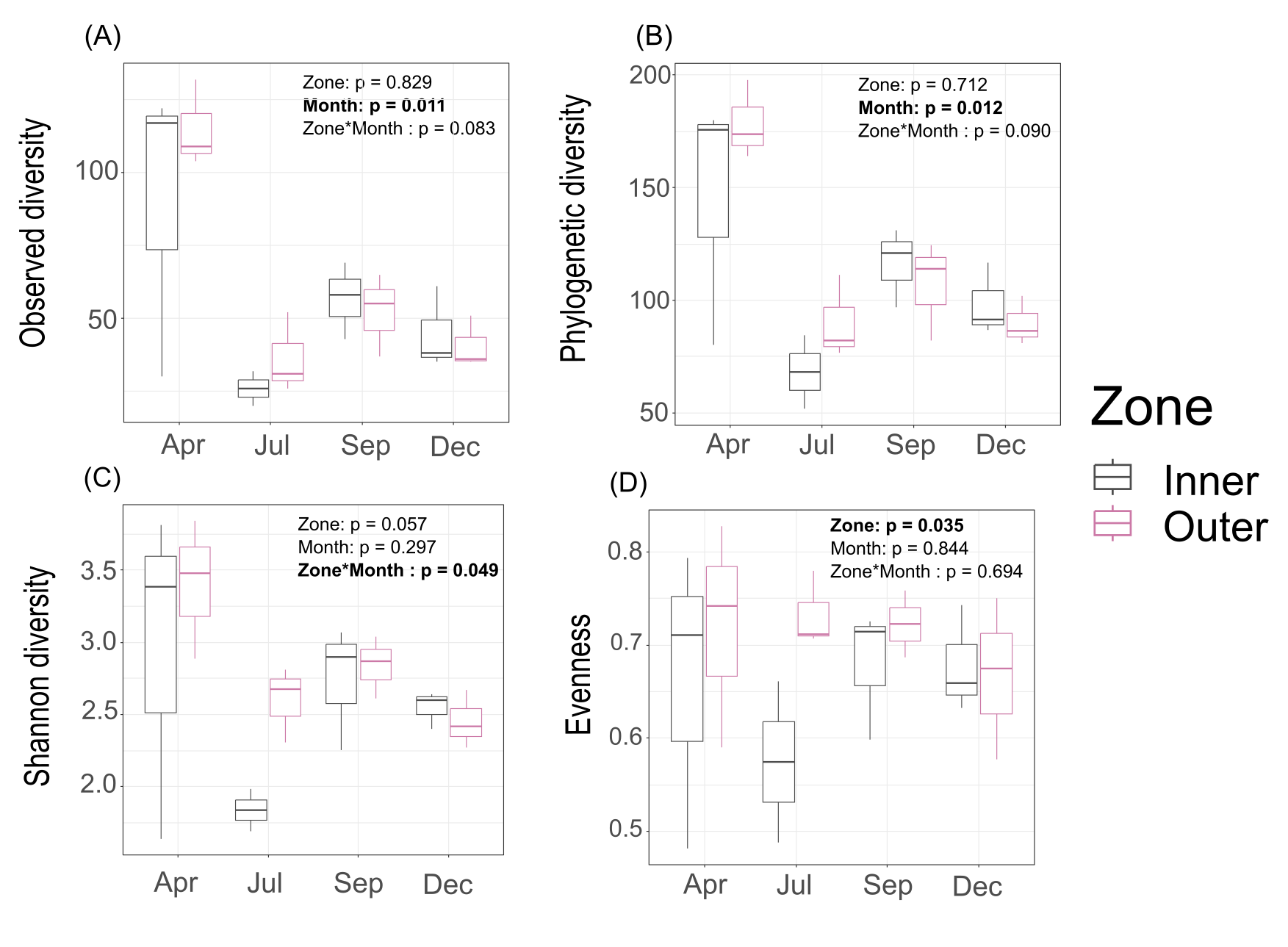

Supplement: Supplemental Information 6 — (A) Observed diversity; (B) phylogenetic diversity; (C) Shannon diversity; (D) evenness. [file peerj-12-17421-s006.png]
