# Supplementary material for: Seasonal dynamics and environmental drivers of tissue and mucus microbiomes in the staghorn coral Acropora pulchra
Source: PeerJ. 2024 May 30;12:e17421. doi: 10.7717/peerj.17421 (PMC11144401; doi:10.7717/peerj.17421)
Supplement: Supplemental Information 7 — All samples were submitted to GenBank under BioProject PRJNA1011454. Collection dates and coordinates are provided for each sample. The tag number represents the unique tag used to identify coral colonies that were repeatedly sampled during the course of the study. Non-chimeric sequences identified by DADA2 are provided for each sample. SRA: Sequence Read Archive. [file peerj-12-17421-s007.docx]

**Supplemental Table 1.** GenBank accession numbers for metabarcoding data included in the analyses. All samples were submitted to GenBank under BioProject PRJNA1011454. Collection dates and coordinates are provided for each sample. The tag number represents the unique tag used to identify coral colonies that were repeatedly sampled during the course of the study. Non-chimeric sequences identified by DADA2 are provided for each sample. SRA: Sequence Read Archive.

| **Biosample Accession** | **SRA Accession** | **Collection Date** | **Coordinates** | **Tag #** | **Sample Type** | **Non-chimeric Sequences** |
| --- | --- | --- | --- | --- | --- | --- |
| SAMN37218957 | SRR25867451 | 2021-04-24 | 13.482 N 144.746 E | 61 | Tissue | 37,952 |
| SAMN37219041 | SRR25867428 | 2021-04-24 | 13.482 N 144.745 E | 61 | Mucus | 250,740 |
| SAMN37218970 | SRR25867350 | 2021-07-01 | 13.482 N 144.746 E | 61 | Tissue | 292,933 |
| SAMN37219054 | SRR25867348 | 2021-07-01 | 13.482 N 144.746 E | 61 | Mucus | 6,091 |
| SAMN37218993 | SRR25867293 | 2021-09-21 | 13.482 N 144.746 E | 61 | Tissue | 49,361 |
| SAMN37219077 | SRR25867292 | 2021-09-21 | 13.482 N 144.746 E | 61 | Mucus | 20,527 |
| SAMN37219015 | SRR25867435 | 2021-12-28 | 13.482 N 144.746 E | 61 | Tissue | 249,543 |
| SAMN37219099 | SRR25867431 | 2021-12-28 | 13.482 N 144.746 E | 61 | Mucus | 8,838 |
| SAMN37218955 | SRR25867301 | 2021-04-24 | 13.482 N 144.746 E | 62 | Tissue | 237,022 |
| SAMN37219039 | SRR25867381 | 2021-04-24 | 13.482 N 144.745 E | 62 | Mucus | 158,651 |
| SAMN37218971 | SRR25867345 | 2021-07-01 | 13.482 N 144.746 E | 62 | Tissue | 216,788 |
| SAMN37219055 | SRR25867344 | 2021-07-01 | 13.482 N 144.746 E | 62 | Mucus | 28,341 |
| SAMN37218994 | SRR25867291 | 2021-09-21 | 13.482 N 144.746 E | 62 | Tissue | 2,764 |
| SAMN37219078 | SRR25867290 | 2021-09-21 | 13.482 N 144.746 E | 62 | Mucus | 111,027 |
| SAMN37219016 | SRR25867433 | 2021-12-28 | 13.482 N 144.746 E | 62 | Tissue | 122,195 |
| SAMN37219100 | SRR25867429 | 2021-12-28 | 13.482 N 144.746 E | 62 | Mucus | 51,753 |
| SAMN37218958 | SRR25867427 | 2021-04-24 | 13.482 N 144.746 E | 63 | Tissue | 166,360 |
| SAMN37219042 | SRR25867409 | 2021-04-24 | 13.482 N 144.744 E | 63 | Mucus | 141,616 |
| SAMN37218972 | SRR25867343 | 2021-07-01 | 13.482 N 144.746 E | 63 | Tissue | 13,377 |
| SAMN37219056 | SRR25867342 | 2021-07-01 | 13.482 N 144.746 E | 63 | Mucus | 9,123 |
| SAMN37218995 | SRR25867389 | 2021-09-21 | 13.482 N 144.746 E | 63 | Tissue | 40,208 |
| SAMN37219079 | SRR25867388 | 2021-09-21 | 13.482 N 144.746 E | 63 | Mucus | 12,034 |
| SAMN37219017 | SRR25867430 | 2021-12-28 | 13.482 N 144.746 E | 63 | Tissue | 107,183 |
| SAMN37219101 | SRR25867425 | 2021-12-28 | 13.482 N 144.745 E | 63 | Mucus | 93,048 |
| SAMN37218952 | SRR25867456 | 2021-04-24 | 13.482 N 144.745 E | 64 | Tissue | 37,509 |
| SAMN37219036 | SRR25867347 | 2021-04-24 | 13.482 N 144.746 E | 64 | Mucus | 145,403 |
| SAMN37218974 | SRR25867339 | 2021-07-01 | 13.482 N 144.745 E | 64 | Tissue | 271,399 |
| SAMN37218996 | SRR25867387 | 2021-09-21 | 13.482 N 144.745 E | 64 | Tissue | 45,210 |
| SAMN37219080 | SRR25867386 | 2021-09-21 | 13.482 N 144.745 E | 64 | Mucus | 105,101 |
| SAMN37219018 | SRR25867426 | 2021-12-28 | 13.482 N 144.745 E | 64 | Tissue | 64,736 |
| SAMN37219102 | SRR25867423 | 2021-12-28 | 13.482 N 144.745 E | 64 | Mucus | 14,999 |
| SAMN37218960 | SRR25867356 | 2021-04-24 | 13.482 N 144.745 E | 65 | Tissue | 43,569 |
| SAMN37219044 | SRR25867353 | 2021-04-24 | 13.482 N 144.744 E | 65 | Mucus | 32,839 |
| SAMN37218975 | SRR25867336 | 2021-07-01 | 13.482 N 144.745 E | 65 | Tissue | 170,389 |
| SAMN37219059 | SRR25867335 | 2021-07-01 | 13.482 N 144.745 E | 65 | Mucus | 89,345 |
| SAMN37218997 | SRR25867385 | 2021-09-21 | 13.482 N 144.745 E | 65 | Tissue | 68,876 |
| SAMN37219081 | SRR25867384 | 2021-09-21 | 13.482 N 144.745 E | 65 | Mucus | 38,654 |
| SAMN37219019 | SRR25867424 | 2021-12-28 | 13.482 N 144.745 E | 65 | Tissue | 23,570 |
| SAMN37219103 | SRR25867422 | 2021-12-28 | 13.482 N 144.745 E | 65 | Mucus | 19,789 |
| SAMN37218954 | SRR25867324 | 2021-04-24 | 13.482 N 144.745 E | 66 | Tissue | 64,089 |
| SAMN37219038 | SRR25867302 | 2021-04-24 | 13.482 N 144.746 E | 66 | Mucus | 205,286 |
| SAMN37218976 | SRR25867334 | 2021-07-01 | 13.482 N 144.745 E | 66 | Tissue | 135,976 |
| SAMN37219060 | SRR25867333 | 2021-07-01 | 13.482 N 144.745 E | 66 | Mucus | 4,400 |
| SAMN37218998 | SRR25867382 | 2021-09-21 | 13.482 N 144.745 E | 66 | Tissue | 76,535 |
| SAMN37218956 | SRR25867380 | 2021-04-24 | 13.482 N 144.744 E | 67 | Tissue | 115,382 |
| SAMN37219040 | SRR25867452 | 2021-04-24 | 13.482 N 144.745 E | 67 | Mucus | 128,529 |
| SAMN37218978 | SRR25867330 | 2021-07-01 | 13.482 N 144.744 E | 67 | Tissue | 92,596 |
| SAMN37219062 | SRR25867329 | 2021-07-01 | 13.482 N 144.744 E | 67 | Mucus | 10,118 |
| SAMN37219000 | SRR25867377 | 2021-09-21 | 13.482 N 144.744 E | 67 | Tissue | 114,596 |
| SAMN37219084 | SRR25867374 | 2021-09-21 | 13.482 N 144.744 E | 67 | Mucus | 58,916 |
| SAMN37219021 | SRR25867418 | 2021-12-28 | 13.482 N 144.744 E | 67 | Tissue | 25,884 |
| SAMN37219105 | SRR25867417 | 2021-12-28 | 13.482 N 144.744 E | 67 | Mucus | 94,909 |
| SAMN37218953 | SRR25867346 | 2021-04-24 | 13.482 N 144.744 E | 68 | Tissue | 62,026 |
| SAMN37218981 | SRR25867323 | 2021-07-01 | 13.482 N 144.744 E | 68 | Tissue | 198,967 |
| SAMN37219065 | SRR25867320 | 2021-07-01 | 13.480 N 144.743 E | 68 | Mucus | 1,440 |
| SAMN37219001 | SRR25867375 | 2021-09-21 | 13.482 N 144.744 E | 68 | Tissue | 308,652 |
| SAMN37219085 | SRR25867371 | 2021-09-21 | 13.482 N 144.744 E | 68 | Mucus | 49,694 |
| SAMN37219022 | SRR25867416 | 2021-12-28 | 13.482 N 144.744 E | 68 | Tissue | 21,117 |
| SAMN37219106 | SRR25867415 | 2021-12-28 | 13.482 N 144.744 E | 68 | Mucus | 30,154 |
| SAMN37218961 | SRR25867455 | 2021-04-24 | 13.482 N 144.744 E | 69 | Tissue | 57,519 |
| SAMN37219045 | SRR25867349 | 2021-04-24 | 13.480 N 144.743 E | 69 | Mucus | 99,525 |
| SAMN37218980 | SRR25867326 | 2021-07-01 | 13.482 N 144.744 E | 69 | Tissue | 51,353 |
| SAMN37219064 | SRR25867322 | 2021-07-01 | 13.482 N 144.744 E | 69 | Mucus | 66,907 |
| SAMN37219002 | SRR25867373 | 2021-09-21 | 13.482 N 144.744 E | 69 | Tissue | 70,306 |
| SAMN37219086 | SRR25867369 | 2021-09-21 | 13.480 N 144.743 E | 69 | Mucus | 45,020 |
| SAMN37219023 | SRR25867414 | 2021-12-28 | 13.482 N 144.744 E | 69 | Tissue | 63,130 |
| SAMN37219107 | SRR25867413 | 2021-12-28 | 13.482 N 144.744 E | 69 | Mucus | 82,328 |
| SAMN37218967 | SRR25867421 | 2021-04-24 | 13.480 N 144.743 E | 72 | Tissue | 133,786 |
| SAMN37219051 | SRR25867410 | 2021-04-24 | 13.480 N 144.743 E | 72 | Mucus | 108,955 |
| SAMN37218989 | SRR25867304 | 2021-07-01 | 13.480 N 144.743 E | 72 | Tissue | 22,657 |
| SAMN37219011 | SRR25867444 | 2021-09-21 | 13.480 N 144.743 E | 72 | Tissue | 55,748 |
| SAMN37219095 | SRR25867440 | 2021-09-21 | 13.480 N 144.743 E | 72 | Mucus | 21,057 |
| SAMN37219032 | SRR25867392 | 2021-12-28 | 13.480 N 144.743 E | 72 | Tissue | 14,659 |
| SAMN37219116 | SRR25867391 | 2021-12-28 | 13.480 N 144.743 E | 72 | Mucus | 80,635 |
| SAMN37218968 | SRR25867399 | 2021-04-24 | 13.480 N 144.743 E | 73 | Tissue | 167,723 |
| SAMN37219052 | SRR25867360 | 2021-04-24 | 13.480 N 144.743 E | 73 | Mucus | 25,735 |
| SAMN37218990 | SRR25867300 | 2021-07-01 | 13.480 N 144.743 E | 73 | Tissue | 41,517 |
| SAMN37219074 | SRR25867299 | 2021-07-01 | 13.480 N 144.743 E | 73 | Mucus | 1,023 |
| SAMN37219012 | SRR25867441 | 2021-09-21 | 13.480 N 144.743 E | 73 | Tissue | 105,278 |
| SAMN37219096 | SRR25867438 | 2021-09-21 | 13.480 N 144.743 E | 73 | Mucus | 38,448 |
| SAMN37219033 | SRR25867390 | 2021-12-28 | 13.480 N 144.743 E | 73 | Tissue | 30,351 |
| SAMN37219117 | SRR25867362 | 2021-12-28 | 13.480 N 144.743 E | 73 | Mucus | 29,716 |
| SAMN37218969 | SRR25867352 | 2021-04-24 | 13.480 N 144.743 E | 74 | Tissue | 126,345 |
| SAMN37219053 | SRR25867351 | 2021-04-24 | 13.480 N 144.743 E | 74 | Mucus | 17,136 |
| SAMN37218991 | SRR25867298 | 2021-07-01 | 13.480 N 144.743 E | 74 | Tissue | 43,890 |
| SAMN37219075 | SRR25867297 | 2021-07-01 | 13.480 N 144.743 E | 74 | Mucus | 1,365 |
| SAMN37219013 | SRR25867439 | 2021-09-21 | 13.480 N 144.743 E | 74 | Tissue | 76,272 |
| SAMN37219034 | SRR25867359 | 2021-12-28 | 13.480 N 144.743 E | 74 | Tissue | 31,642 |
| SAMN37219118 | SRR25867358 | 2021-12-28 | 13.480 N 144.743 E | 74 | Mucus | 111,241 |
| SAMN37218964 | SRR25867294 | 2021-04-24 | 13.480 N 144.743 E | 75 | Tissue | 125,093 |
| SAMN37219048 | SRR25867383 | 2021-04-24 | 13.480 N 144.743 E | 75 | Mucus | 8,982 |
| SAMN37218985 | SRR25867313 | 2021-07-01 | 13.480 N 144.743 E | 75 | Tissue | 362,795 |
| SAMN37219069 | SRR25867312 | 2021-07-01 | 13.480 N 144.743 E | 75 | Mucus | 20,478 |
| SAMN37219007 | SRR25867361 | 2021-09-21 | 13.480 N 144.743 E | 75 | Tissue | 43,557 |
| SAMN37219091 | SRR25867449 | 2021-09-21 | 13.480 N 144.743 E | 75 | Mucus | 97,566 |
| SAMN37219028 | SRR25867401 | 2021-12-28 | 13.480 N 144.743 E | 75 | Tissue | 15,022 |
| SAMN37219112 | SRR25867400 | 2021-12-28 | 13.480 N 144.743 E | 75 | Mucus | 18,200 |
| SAMN37218965 | SRR25867372 | 2021-04-24 | 13.480 N 144.743 E | 76 | Tissue | 82,674 |
| SAMN37219049 | SRR25867454 | 2021-04-24 | 13.480 N 144.743 E | 76 | Mucus | 91,676 |
| SAMN37218986 | SRR25867311 | 2021-07-01 | 13.480 N 144.743 E | 76 | Tissue | 95,065 |
| SAMN37219070 | SRR25867310 | 2021-07-01 | 13.480 N 144.743 E | 76 | Mucus | 1,641 |
| SAMN37219008 | SRR25867450 | 2021-09-21 | 13.480 N 144.743 E | 76 | Tissue | 193,058 |
| SAMN37219092 | SRR25867447 | 2021-09-21 | 13.480 N 144.743 E | 76 | Mucus | 99,413 |
| SAMN37219029 | SRR25867398 | 2021-12-28 | 13.480 N 144.743 E | 76 | Tissue | 20,489 |
| SAMN37219113 | SRR25867397 | 2021-12-28 | 13.480 N 144.743 E | 76 | Mucus | 28,788 |
| SAMN37218966 | SRR25867432 | 2021-04-24 | 13.480 N 144.743 E | 77 | Tissue | 67,824 |
| SAMN37219050 | SRR25867443 | 2021-04-24 | 13.480 N 144.743 E | 77 | Mucus | 274,535 |
| SAMN37218987 | SRR25867309 | 2021-07-01 | 13.480 N 144.743 E | 77 | Tissue | 85,978 |
| SAMN37219071 | SRR25867308 | 2021-07-01 | 13.480 N 144.743 E | 77 | Mucus | 2,408 |
| SAMN37219009 | SRR25867448 | 2021-09-21 | 13.480 N 144.743 E | 77 | Tissue | 45,625 |
| SAMN37219030 | SRR25867396 | 2021-12-28 | 13.480 N 144.743 E | 77 | Tissue | 25,829 |
| SAMN37219114 | SRR25867395 | 2021-12-28 | 13.480 N 144.743 E | 77 | Mucus | 63,828 |
| SAMN37218962 | SRR25867338 | 2021-04-24 | 13.480 N 144.742 E | 78 | Tissue | 62,281 |
| SAMN37219046 | SRR25867327 | 2021-04-24 | 13.480 N 144.743 E | 78 | Mucus | 83,536 |
| SAMN37218982 | SRR25867321 | 2021-07-01 | 13.480 N 144.743 E | 78 | Tissue | 29,620 |
| SAMN37219066 | SRR25867319 | 2021-07-01 | 13.480 N 144.743 E | 78 | Mucus | 1,311 |
| SAMN37219003 | SRR25867370 | 2021-09-21 | 13.480 N 144.743 E | 78 | Tissue | 58,969 |
| SAMN37219087 | SRR25867367 | 2021-09-21 | 13.480 N 144.743 E | 78 | Mucus | 43,578 |
| SAMN37219024 | SRR25867412 | 2021-12-28 | 13.480 N 144.743 E | 78 | Tissue | 20,544 |
| SAMN37219108 | SRR25867411 | 2021-12-28 | 13.480 N 144.743 E | 78 | Mucus | 90,894 |
| SAMN37218963 | SRR25867316 | 2021-04-24 | 13.480 N 144.742 E | 79 | Tissue | 344,464 |
| SAMN37219047 | SRR25867305 | 2021-04-24 | 13.480 N 144.743 E | 79 | Mucus | 562,259 |
| SAMN37219004 | SRR25867368 | 2021-09-21 | 13.480 N 144.743 E | 79 | Tissue | 68,656 |
| SAMN37219088 | SRR25867365 | 2021-09-21 | 13.480 N 144.743 E | 79 | Mucus | 28,484 |
| SAMN37219025 | SRR25867407 | 2021-12-28 | 13.480 N 144.743 E | 79 | Tissue | 44,474 |
| SAMN37219109 | SRR25867406 | 2021-12-28 | 13.480 N 144.743 E | 79 | Mucus | 141,825 |
| SAMN37218959 | SRR25867408 | 2021-04-24 | 13.480 N 144.742 E | 80 | Tissue | 85,968 |
| SAMN37219043 | SRR25867357 | 2021-04-24 | 13.482 N 144.744 E | 80 | Mucus | 117,351 |
| SAMN37218983 | SRR25867318 | 2021-07-01 | 13.480 N 144.743 E | 80 | Tissue | 157,065 |
| SAMN37219067 | SRR25867317 | 2021-07-01 | 13.480 N 144.743 E | 80 | Mucus | 1,497 |
| SAMN37219005 | SRR25867366 | 2021-09-21 | 13.480 N 144.743 E | 80 | Tissue | 5,316 |
| SAMN37219026 | SRR25867405 | 2021-12-28 | 13.480 N 144.743 E | 80 | Tissue | 64,591 |
| SAMN37219110 | SRR25867404 | 2021-12-28 | 13.480 N 144.743 E | 80 | Mucus | 84,938 |
| SAMN37239985 | SRR25884572 | 2021-04-24 | 13.480 N 144.743 E | N/A | Seawater | 275,093 |
| SAMN37239986 | SRR25884571 | 2021-04-24 | 13.480 N 144.743 E | N/A | Seawater | 53,670 |
| SAMN37239987 | SRR25884584 | 2021-04-24 | 13.480 N 144.743 E | N/A | Seawater | 472,024 |
| SAMN37239988 | SRR25884579 | 2021-04-24 | 13.482 N 144.746 E | N/A | Seawater | 364,150 |
| SAMN37239989 | SRR25884578 | 2021-04-24 | 13.482 N 144.745 E | N/A | Seawater | 169,642 |
| SAMN37239990 | SRR25884577 | 2021-04-24 | 13.482 N 144.744 E | N/A | Seawater | 145,361 |
| SAMN37239992 | SRR25884591 | 2021-07-01 | 13.480 N 144.743 E | N/A | Seawater | 74,097 |
| SAMN37239993 | SRR25884590 | 2021-07-01 | 13.480 N 144.743 E | N/A | Seawater | 6,731 |
| SAMN37239994 | SRR25884589 | 2021-07-01 | 13.482 N 144.746 E | N/A | Seawater | 7,804 |
| SAMN37239995 | SRR25884588 | 2021-07-01 | 13.482 N 144.745 E | N/A | Seawater | 14,397 |
| SAMN37239996 | SRR25884587 | 2021-07-01 | 13.482 N 144.744 E | N/A | Seawater | 8,035 |
| SAMN37239997 | SRR25884586 | 2021-09-21 | 13.480 N 144.743 E | N/A | Seawater | 15,380 |
| SAMN37239998 | SRR25884585 | 2021-09-21 | 13.480 N 144.743 E | N/A | Seawater | 42,093 |
| SAMN37239999 | SRR25884583 | 2021-09-21 | 13.480 N 144.743 E | N/A | Seawater | 11,884 |
| SAMN37240000 | SRR25884582 | 2021-09-21 | 13.482 N 144.746 E | N/A | Seawater | 11,389 |
| SAMN37240001 | SRR25884581 | 2021-09-21 | 13.482 N 144.745 E | N/A | Seawater | 4,159 |
| SAMN37240002 | SRR25884580 | 2021-09-21 | 13.482 N 144.744 E | N/A | Seawater | 5,448 |
| SAMN37240003 | SRR25884576 | 2021-12-28 | 13.480 N 144.743 E | N/A | Seawater | 67,581 |
| SAMN37240004 | SRR25884575 | 2021-12-28 | 13.480 N 144.743 E | N/A | Seawater | 16,166 |
| SAMN37240005 | SRR25884574 | 2021-12-28 | 13.480 N 144.743 E | N/A | Seawater | 26,744 |
| SAMN37240006 | SRR25884573 | 2021-12-28 | 13.482 N 144.746 E | N/A | Seawater | 52,967 |
| SAMN37240007 | SRR25884570 | 2021-12-28 | 13.482 N 144.745 E | N/A | Seawater | 16,952 |
| SAMN37240008 | SRR25884569 | 2021-12-28 | 13.482 N 144.744 E | N/A | Seawater | 48,214 |
