# Supplementary material for: Seasonal dynamics and environmental drivers of tissue and mucus microbiomes in the staghorn coral Acropora pulchra
Source: PeerJ. 2024 May 30;12:e17421. doi: 10.7717/peerj.17421 (PMC11144401; doi:10.7717/peerj.17421)
Supplement: Supplemental Information 8 — Diversity comparisons for overall, tissue, and mucus compartments were made using a Kruskal-Wallis Chi2 test. Because seawater diversity was normally distributed an analysis of variance (ANOVA) was used for seawater comparisons. Significant results (p(perm) <0.05) are highlighted in bold. [file peerj-12-17421-s008.docx]

**Supplemental Table 2.** Shannon diversity comparisons among samples from distinct coral compartments (tissue, mucus and seawater), month (April, July, September and December) and zone (in versus out). Diversity comparisons for overall, tissue, and mucus compartments were made using a Kruskal-Wallis Chi2 test. Because seawater diversity was normally distributed an analysis of variance (ANOVA) was used for seawater comparisons. Significant results (*p*(perm) <0.05) are highlighted in bold.

**Overall**

| Source of Variation  Interactions | *df* | X^2^ | *p(*perm) |
| --- | --- | --- | --- |
| Compartment | 2 | 63.138 | **< 0.001** |
| Zone | 1 | 1.0288 | 0.310 |
| Month | 3 | 7.6118 | 0.055 |
| Zone:Month | 7 | 9.2215 | 0.237 |
| Compartment:Zone | 5 | 64.776 | **< 0.001** |
| Compartment:Month | 11 | 73.897 | **< 0.001** |
| Compartment:Zone:Month | 23 | 86.204 | **< 0.001** |

**Tissue**

| Source of Variation  Interactions | *df* | X^2^ | *p(*perm) |
| --- | --- | --- | --- |
| Zone | 1 | 0.018247 | 0.893 |
| Month | 3 | 6.1154 | 0.106 |
| Zone:Month | 7 | 13.53 | 0.060 |

**Mucus**

| Source of Variation  Interactions | *df* | X^2^ | *p(*perm) |
| --- | --- | --- | --- |
| Zone | 1 | 1.2883 | 0.256 |
| Month | 3 | 8.567 | **0.036** |
| Zone:Month | 7 | 14.507 | **0.043** |

**Seawater**

| Source of Variation  Interactions | *df* | F value | *p(*perm) |
| --- | --- | --- | --- |
| Zone | 1 | 0.087 | 0.771 |
| Month | 1 | 2.967 | 0.100 |
| Zone:Month | 1 | 0.970 | 0.337 |
