# Supplementary material for: Seasonal dynamics and environmental drivers of tissue and mucus microbiomes in the staghorn coral Acropora pulchra
Source: PeerJ. 2024 May 30;12:e17421. doi: 10.7717/peerj.17421 (PMC11144401; doi:10.7717/peerj.17421)
Supplement: Supplemental Information 9 — Evenness comparisons for overall, tissue, and mucus compartments were made using a Kruskal-Wallis Chi2 test. Evenness comparisons for seawater were made using an analysis of variance (ANOVA), as evenness values were normally distributed for seawater. Significant results (p(perm) <0.05) are highlighted in bold. [file peerj-12-17421-s009.docx]

**Supplemental Table 3.** Microbial evenness Shannon diversity comparisons among samples from distinct coral compartments (tissue, mucus and seawater), month (April, July, September and December) and zone (in versus out). Evenness comparisons for overall, tissue, and mucus compartments were made using a Kruskal-Wallis Chi2 test. Evenness comparisons for seawater were made using an analysis of variance (ANOVA), as evenness values were normally distributed for seawater. Significant results (*p*(perm) <0.05) are highlighted in bold.

**Overall**

| Source of Variation  Interactions | *df* | X^2^ | *p(*perm) |
| --- | --- | --- | --- |
| Compartment | 2 | 64.665 | **< 0.001** |
| Zone | 1 | 0.282 | 0.596 |
| Month | 3 | 2.012 | 0.570 |
| Zone:Month | 7 | 4.000 | 0.780 |
| Compartment:Zone | 5 | 65.649 | **< 0.001** |
| Compartment:Month | 11 | 76.174 | **< 0.001** |
| Compartment:Zone:Month | 23 | 84.665 | **< 0.001** |

**Tissue**

| Source of Variation  Interactions | *df* | X^2^ | *p(*perm) |
| --- | --- | --- | --- |
| Zone | 1 | 0.004 | 0.949 |
| Month | 3 | 6.768 | 0.080 |
| Zone:Month | 7 | 15.138 | **0.034** |

**Mucus**

| Source of Variation  Interactions | *df* | X^2^ | *p(*perm) |
| --- | --- | --- | --- |
| Zone | 1 | 0.005 | 0.946 |
| Month | 3 | 11.970 | **0.008** |
| Zone:Month | 7 | 15.385 | 0.314 |

**Seawater**

| Source of Variation  Interactions | *df* | F value | *p(*perm) |
| --- | --- | --- | --- |
| Zone | 1 | 2.362 | 0.139 |
| Month | 1 | 0.040 | 0.844 |
| Zone:Month | 7 | 0.671 | 0.694 |
