# Supplementary material for: Seasonal dynamics and environmental drivers of tissue and mucus microbiomes in the staghorn coral Acropora pulchra
Source: PeerJ. 2024 May 30;12:e17421. doi: 10.7717/peerj.17421 (PMC11144401; doi:10.7717/peerj.17421)
Supplement: Supplemental Information 10 — PD comparisons for overall, tissue, and seawater compartments were made using a Kruskal-Wallis Chi2 test. Because mucus diversity had a normal distribution, an analysis of variance (ANOVA) was used. Significant results (p(perm) <0.05) are highlighted in bold. [file peerj-12-17421-s010.docx]

**Supplemental Table 4.** Faith’s Phylogenetic Diversity (PD) comparisons among samples from distinct compartments (seawater, mucus and tissue), month (April, July, September and December) and zone (in versus out). PD comparisons for overall, tissue, and seawater compartments were made using a Kruskal-Wallis Chi2 test. Because mucus diversity had a normal distribution, an analysis of variance (ANOVA) was used. Significant results (*p*(perm) <0.05) are highlighted in bold.

**Overall**

| Source of Variation  Interactions | *df* | X^2^ | *p(*perm) |
| --- | --- | --- | --- |
| Compartment | 2 | 15.000 | **< 0.001** |
| Zone | 1 | 0.922 | 0.374 |
| Month | 3 | 27.109 | **< 0.001** |
| Zone:Month | 7 | 31.658 | **< 0.001** |
| Compartment:Zone | 5 | 16.057 | **0.002** |
| Compartment:Month | 11 | 61.849 | **< 0.001** |
| Compartment:Zone:Month | 23 | 72.317 | **< 0.001** |

**Tissue**

| Source of Variation  Interactions | *df* | X^2^ | *p(*perm) |
| --- | --- | --- | --- |
| Zone | 1 | 0.164 | 0.685 |
| Month | 3 | 11.218 | **0.011** |
| Zone:Month | 7 | 16.622 | **0.020** |

**Mucus**

| Source of Variation  Interactions | *df* | F value | *p(*perm) |
| --- | --- | --- | --- |
| Zone | 1 | 0.781 | 0.380 |
| Month | 1 | 2.350 | 0.130 |
| Zone:Month | 7 | 8.105 | **< 0.001** |

**Seawater**

| Source of Variation  Interactions | *df* | X^2^ | *p(*perm) |
| --- | --- | --- | --- |
| Zone | 1 | 0.136 | 0.712 |
| Month | 3 | 10.822 | **0.012** |
| Zone:Month | 7 | 12.337 | 0.090 |
