# Supplementary material for: Seasonal dynamics and environmental drivers of tissue and mucus microbiomes in the staghorn coral Acropora pulchra
Source: PeerJ. 2024 May 30;12:e17421. doi: 10.7717/peerj.17421 (PMC11144401; doi:10.7717/peerj.17421)
Supplement: Supplemental Information 13 — Significant results (p(perm) <0.05) are highlighted in bold. [file peerj-12-17421-s013.docx]

**Supplemental Table 7.** Permutational multivariate analysis of variance (PERMANOVA) table for beta diversity comparisons among microbial communities from distinct coral compartments (seawater, mucus and tissue), month (April, July, September and December) and zone (in *versus* out). Significant results (*p*(perm) <0.05) are highlighted in bold.

**Overall**

| Source of Variation  Interactions | *df* | F value | *p(*perm) |
| --- | --- | --- | --- |
| Compartment | 2 | 15.035 | **< 0.001** |
| Zone | 1 | 2.198 | 0.064 |
| Month | 1 | 3.870 | **0.009** |
| Zone:Month | 1 | 3.097 | **0.019** |
| Compartment:Zone | 2 | 1.429 | 0.150 |
| Compartment:Month | 2 | 2.900 | **0.004** |
| Compartment:Zone:Month | 2 | 1.031 | 0.418 |

**Tissue**

| Source of Variation  Interactions | *df* | F value | *p(*perm) |
| --- | --- | --- | --- |
| Zone | 1 | 4.943 | **0.002** |
| Month | 1 | 1.418 | 0.244 |
| Zone:Month | 1 | 4.508 | **0.003** |

**Mucus**

| Source of Variation  Interactions | *df* | F value | *p(*perm) |
| --- | --- | --- | --- |
| Zone | 1 | 1.033 | 0.353 |
| Month | 1 | 7.213 | **< 0.001** |
| Zone:Month | 1 | 1.950 | 0.074 |

**Seawater**

| Source of Variation  Interactions | *df* | F value | *p(*perm) |
| --- | --- | --- | --- |
| Zone | 1 | 0.537 | 0.827 |
| Month | 1 | 1.556 | 0.160 |
| Zone:Month | 1 | 0.291 | 0.982 |
