# Supplementary material for: Seasonal dynamics and environmental drivers of tissue and mucus microbiomes in the staghorn coral Acropora pulchra
Source: PeerJ. 2024 May 30;12:e17421. doi: 10.7717/peerj.17421 (PMC11144401; doi:10.7717/peerj.17421)
Supplement: Supplemental Information 14 — Significant results (p(perm) <0.05) are highlighted in bold. [file peerj-12-17421-s014.docx]

**Supplemental Table 8.** Post-hoc permutational multivariate analysis of variance (PERMANOVA) table for pairwise beta diversity comparisons among microbial communities between individual compartments (seawater, coral mucus and coral tissue), and individual months (May, July, September and December). Significant results (*p*(perm) <0.05) are highlighted in bold.

**Overall**

| Source of Variation  Interactions | *df* | F value | *p(*perm) |
| --- | --- | --- | --- |
| Tissue vs. Mucus | 1 | 18.709 | **0.003** |
| Tissue vs. Seawater | 1 | 14.378 | **0.003** |
| Mucus vs. Seawater | 1 | 3.458 | **0.024** |
| Apr vs. Jul | 1 | 2.952 | 0.090 |
| Apr vs. Sep | 1 | 2.540 | 0.138 |
| Apr vs. Dec | 1 | 4.615 | **0.006** |
| Jul vs. Sep | 1 | 4.508 | **0.006** |
| Jul vs. Dec | 1 | 5.781 | **0.012** |
| Sep vs. Dec | 1 | 2.035 | 0.390 |

**Tissue**

| Source of Variation  Interactions | *df* | Pseudo-*F* | *p(*perm) |
| --- | --- | --- | --- |
| Apr vs. Jul | 1 | 1.153 | 1.000 |
| Apr vs. Sep | 1 | 0.577 | 1.000 |
| Apr vs. Dec | 1 | 2.598 | 0.443 |
| Jul vs. Sep | 1 | 2.103 | 0.527 |
| Jul vs. Dec | 1 | 1.272 | 1.000 |
| Sep vs. Dec | 1 | 3.223 | 0.108 |

**Mucus**

| Source of Variation  Interactions | *df* | Pseudo-*F* | *p(*perm) |
| --- | --- | --- | --- |
| Apr vs. Jul | 1 | 11.594 | **0.006** |
| Apr vs. Sep | 1 | 3.299 | **0.006** |
| Apr vs. Dec | 1 | 6.010 | **0.006** |
| Jul vs. Sep | 1 | 9.608 | **0.006** |
| Jul vs. Dec | 1 | 16.631 | **0.006** |
| Sep vs. Dec | 1 | 0.822 | 1.000 |

**Seawater**

| Source of Variation  Interactions | *df* | F value | *p(*perm) |
| --- | --- | --- | --- |
| Apr vs. Jul | 1 | 1.379 | 1.000 |
| Apr vs. Sep | 1 | 1.715 | 1.000 |
| Apr vs. Dec | 1 | 2.557 | 0.360 |
| Jul vs. Sep | 1 | 3.017 | 0.108 |
| Jul vs. Dec | 1 | 2.881 | 0.114 |
| Sep vs. Dec | 1 | 8.461 | **0.018** |
