# Supplementary material for: Seasonal dynamics and environmental drivers of tissue and mucus microbiomes in the staghorn coral Acropora pulchra
Source: PeerJ. 2024 May 30;12:e17421. doi: 10.7717/peerj.17421 (PMC11144401; doi:10.7717/peerj.17421)
Supplement: Supplemental Information 18 — Archive containing the R scripts used in data analyses. Tables with environmental data are included in the archive as well. [file peerj-12-17421-s018.zip › README.docx]

Scripts used for analysis of environmental data, microbiome diversity and composition, and the construction of the structural equation model (SEM).

The SEM relies on data and products found in the environmental data and microbiome data analysis folders.
