# Supplementary material for: Seasonal dynamics and environmental drivers of tissue and mucus microbiomes in the staghorn coral Acropora pulchra
Source: PeerJ. 2024 May 30;12:e17421. doi: 10.7717/peerj.17421 (PMC11144401; doi:10.7717/peerj.17421)
Supplement: Supplementary file 28 [file peerj-12-17421-s011.odt]

Supplemental Table 5. Relative abundances of six most abundant phyla found in coral tissue, coral mucus, and seawater. Relative abundance was calculated as percentage of sequence tags associated with each phylum.

| Phylum | Overall % | Tissue % | Mucus % | Seawater % |
| --- | --- | --- | --- | --- |
| Actinobacteriota | 2.6 | 1.1 | 4.5 | 2.3 |
| Bacteroidota | 5.9 | 1.6 | 6.7 | 19.3 |
| Cyanobacteria | 2.0 | 0.4 | 2.2 | 7.3 |
| Firmicutes | 2.3 | 1.2 | 3.9 | 1.6 |
| Proteobacteria | 76.5 | 83.7 | 72.4 | 63.3 |
| Verrucomicrobiota | 9.9 | 11.7 | 9.3 | 5.2 |
