# Supplementary material for: Seasonal dynamics and environmental drivers of tissue and mucus microbiomes in the staghorn coral Acropora pulchra
Source: PeerJ. 2024 May 30;12:e17421. doi: 10.7717/peerj.17421 (PMC11144401; doi:10.7717/peerj.17421)
Supplement: Supplementary file 29 [file peerj-12-17421-s012.odt]

Supplemental Table 6. Relative abundances of 11 most abundant families found in all compartments, in coral tissue, in coral mucus, and in seawater. Relative abundance was calculated as percentage of sequence tags associated with each family.

| Family | Overall % | Tissue % | Mucus % | Seawater % |
| --- | --- | --- | --- | --- |
| Alteromonadaceae | 1.4 | 0.5 | 1.6 | 4.2 |
| Chitinophageacae | 3.2 | 0.9 | 5.0 | 6.0 |
| Comamonadaceae | 6.8 | 1.6 | 13.6 | 5.1 |
| Cryomorphaceae | 0.8 | 0.1 | 0.4 | 4.7 |
| Cyanobiaceae | 1.7 | 0.3 | 1.6 | 7.3 |
| Endozoicomonadaceae | 51.0 | 76.2 | 31.1 | 23.8 |
| Moraxellaceae | 6.2 | 2.5 | 11.3 | 4.3 |
| Pseudomonadaceae | 1.1 | 0.2 | 2.5 | 0.5 |
| Rhodobacteraceae | 1.3 | 0.1 | 1.2 | 6.3 |
| Simkaniaceae | 9.8 | 11.8 | 9.1 | 4.8 |
| Sphingomonadaceae | 2.1 | 0.5 | 3.8 | 2.8 |
