# Supplementary material for: Seasonal dynamics and environmental drivers of tissue and mucus microbiomes in the staghorn coral Acropora pulchra
Source: PeerJ. 2024 May 30;12:e17421. doi: 10.7717/peerj.17421 (PMC11144401; doi:10.7717/peerj.17421)
Supplement: Supplementary file 30 [file peerj-12-17421-s015.odt]

Supplemental Table 9. Analysis of similarities (ANOSIM) comparing microbial communities of the different coral compartments (seawater, coral mucus and coral tissue). Samples were separated into different seasons (wet vs. dry) and reef zones (inner vs. outer) to analyze within group similarities.

Overall

| Source of Variation Interactions | ANOSIM statistic R | p(perm) |
| --- | --- | --- |
| Season | 0.034 | 0.005 |
| Zone | 0.009 | 0.113 |
| Dry season: Zone | 0.130 | 0.001 |
| Wet season: Zone | -0.012 | 0.734 |
| Inner zone: Season | 0.111 | < 0.001 |
| Outer zone: Season | 0.047 | 0.022 |

Tissue

| Source of Variation Interactions | ANOSIM statistic R | p(perm) |
| --- | --- | --- |
| Season | -0.001 | 0.382 |
| Zone | 0.403 | 0.382 |
| Dry Season: Zone | 0.352 | 0.002 |
| Wet season: Zone | -0.009 | 0.523 |
| Inner zone: Season | 0.161 | 0.002 |
| Outer zone: Season | 0.169 | 0.010 |

Mucus

| Source of Variation Interactions | ANOSIM statistic R | p(perm) |
| --- | --- | --- |
| Season | 0.315 | < 0.001 |
| Zone | -0.021 | 0.926 |
| Dry season: Zone | -0.026 | 0.774 |
| Wet season: Zone | 0.007 | 0.270 |
| Inner zone: Season | 0.309 | 0.005 |
| Outer zone: Season | 0.417 | < 0.001 |

Seawater

| Source of Variation Interactions | ANOSIM statistic R | p(perm) |
| --- | --- | --- |
| Season | 0.056 | 0.180 |
| Zone | -0.013 | 0.459 |
| Dry season: Zone | 0.008 | 0.366 |
| Wet season: Zone | -0.109 | 0.856 |
| Inner zone: Season | -0.072 | 0.652 |
| Outer zone: Season | 0.044 | 0.257 |
